# Genomic profiling using array comparative genomic hybridization define distinct subtypes of diffuse large b-cell lymphoma: a review of the literature

**DOI:** 10.1186/1756-8722-5-54

**Published:** 2012-09-11

**Authors:** Carlos A Tirado, Weina Chen, Rolando García, Kelly A Kohlman, Nagesh Rao

**Affiliations:** 1 Department of Pathology & Laboratory Medicine UCLA - David Geffen UCLA, School of Medicine, Los Angeles, USA; 2 Ameripath/Quest Diagnostics, Dallas, TX, USA; 3 Department of Pathology, The UT Southwestern Medical Center, Clinical Cytogenetics, Dallas, USA; 4 Clinical Cytogenetics, The University of Texas Health Science Center at San Antonio, San Antonio, TX, USA

**Keywords:** DLBCL, Array CGH, Genomic profiles

## Abstract

Diffuse large B-cell lymphoma (DLBCL) is the most common type of non-Hodgkin Lymphoma comprising of greater than 30% of adult non-Hodgkin Lymphomas. DLBCL represents a diverse set of lymphomas, defined as diffuse proliferation of large B lymphoid cells. Numerous cytogenetic studies including karyotypes and fluorescent in situ hybridization (FISH), as well as morphological, biological, clinical, microarray and sequencing technologies have attempted to categorize DLBCL into morphological variants, molecular and immunophenotypic subgroups, as well as distinct disease entities. Despite such efforts, most lymphoma remains undistinguishable and falls into DLBCL, not otherwise specified (DLBCL-NOS). The advent of microarray-based studies (chromosome, RNA, gene expression, etc) has provided a plethora of high-resolution data that could potentially facilitate the finer classification of DLBCL. This review covers the microarray data currently published for DLBCL. We will focus on these types of data; 1) array based CGH; 2) classical CGH; and 3) gene expression profiling studies. The aims of this review were three-fold: (1) to catalog chromosome loci that are present in at least 20% or more of distinct DLBCL subtypes; a detailed list of gains and losses for different subtypes was generated in a table form to illustrate specific chromosome loci affected in selected subtypes; (2) to determine common and distinct copy number alterations among the different subtypes and based on this information, characteristic and similar chromosome loci for the different subtypes were depicted in two separate chromosome ideograms; and, (3) to list re-classified subtypes and those that remained indistinguishable after review of the microarray data. To the best of our knowledge, this is the first effort to compile and review available literatures on microarray analysis data and their practical utility in classifying DLBCL subtypes.

Although conventional cytogenetic methods such as Karyotypes and FISH have played a major role in classification schemes of lymphomas, better classification models are clearly needed to further understanding the biology, disease outcome and therapeutic management of DLBCL. In summary, microarray data reviewed here can provide better subtype specific classifications models for DLBCL.

## Introduction

Diffuse large B-cell lymphoma (DLBCL) is the most frequent non-Hodgkin lymphoma comprising of greater than 30% of adult non-Hodgkin lymphomas in the West, and an even higher percent in developing countries [1]. DLBCL has traditionally been defined as a diffuse proliferation of lymphoid neoplasm in which the nucleus is equal to or exceeds the size of a normal macrophage nucleus [2]. It is clinically heterogeneous and includes a wide spectrum of lymphoid neoplasms.

Many morphological, biological, cytogenetics and clinical studies have attempted to subdivide DLBCL into morphological variants, molecular, immunophenotypic subgroups, and distinct disease entities. While progress has been made in the recent years, many of the cases remain biologically heterogeneous [3,4]. In fact, the most recent World Health Organization (WHO) classification published in 2008 groups most of these lymphomas into the category of DLBCL, not otherwise specified (DLBCL-NOS) [4].

One recent microarray technology applied to distinguish between DLBCL subtypes is array based comparative genomic hybridization (aCGH). Array-based CGH provides high resolution genome wide measurement of DNA copy number alterations highlighting patterns of deletions and amplifications. An additional advantage of array-based CGH is the assessment of allelic ratio or loss of heterozygosity. Here, an assessment of the relative intensity for each of the alleles can be determined. However; one limitation is the inability to detect balanced chromosome translocations. Nonetheless, aCGH can facilitate new construct classifiers for DLBCL subtypes. Other key technology worth mentioning that may provide classification models for DLBCL include single nucleotide polymorphism (SNP) arrays. Indeed, in recent years a number of SNP array studies have been reported that list characteristic SNP in hematolymphoid neoplasms [5-9].

An alternative microarray technology that has been extensively used in the classification of DLBCL is gene expression profiling. Studies using gene expression profiling have stratified DLBCL into favorable and unfavorable groups i.e., the germinal center B-cell like (GCB) and the activated B-cell like (ABC) DLBCL respectively [1,10]. The ABC type expresses genes that are distinctive of activated B-cells and plasma cells with a poor clinical outcome (30% 5-year survival rate), whereas the GCB subtype expresses a molecular signature of normal germinal center B-cells with a more favorable overall survival (59% 5-year survival rate) [11-14]. Amplifications of the *REL* loci, *BCL-2* translocations and hypermutations of the immunoglobulins loci are typical of the GCB-DLBCL subtype [1,11,15]. In contrast, constitutive activation of the nuclear factor *kB* pathway is a distinctive feature of both the ABC and primary mediastinal B-cell lymphoma (PMBL) subtype [16-19]. A third type also identified from others by molecular profiling is PMBL with frequent amplifications at 2p and 9p corresponding to *JAK-2* and *REL* respectively with a 64% 5-year survival rate [2,11,13,20,21]. Further studies with high resolution array comparative genomic hybridization (aCGH) have revealed recurrent copy number alterations (CNA), as well as prognostic indicators in a number of DLBCL subtypes [22-27], for example, in a recent high resolution CGH study, CNA resistant to rituximab, cyclophosphamide, doxorubicin, vincristine, and prednisone (R-CHOP) therapy included amplifications of 1p36.13, 1q42.3, 3p21.31, 7q11.23 and 16p13.3, as well as losses at 9p21.3 and 14p21.31 in DLBCL. Various reasons have been proposed for the various CNA just mentioned above and immuno-chemoresistance. These include: faulty p53/INK/ARF functioning caused by 9p21.3 deletions seems to disrupt p53 induced apoptosis, up regulation of various target genes in the nuclear factor *kB* pathway due to copy number gain of *MAPKAPK3* at 3p21.31 leading to nuclear factor *kB* activation and consequently resulting in high expression of various apoptotic inhibiting genes [28-31] and copy number gains at 16p13.3 resulting in overexpression of *ABCA3*, which has been implicated as a likely cause of drug resistance by driving the flow of drugs out of the cell [32].

Despite these efforts to subdivide DLBCL, the relationship between the different classification schemes has not been adequately studied. In this review, we explored CNA linked to well-defined WHO subtypes of DLBCL and compared CNA across the various DLBCL subtypes.

## Review of the literature

### Diffuse large B-cell lymphoma, not otherwise specified (NOS)

DLBCL NOS consists of all DLBCL cases that do not fit into one of the other specific subtypes or disease entities [1]. Since the NOS subgroup continues to exist as an indistinct set of DLBCL, the exact associated aberrations are therefore more difficult to define. A study carried out by Pasqualucci et al. [33] revealed aberrant somatic hypermutations targeting multiple genetic loci including *PIMI, MYC, RHOH/TTF*, and *PAX*. Moreover, abnormalities of band region 3q27, t(14;18) translocations and complex karyotypes are commonly seen in this subtype.

#### Array CGH and gene expression profiles identify molecular subtypes

In 2008, Lenz et al. [2] analyzed 203 DLBCL samples by high resolution array CGH and gene expression profile to investigate the 3 molecular subtypes of DLBCL. Aberrations most characteristic of ABC DLBCL included trisomy 3, deletion of chromosome arm 6q, deletions/duplications of 18q, deletion of *INK4a/ARF* tumor suppressor locus on chromosome 9, and gain-amplification of a 9-megabite (Mb) region on chromosome 19q. Trisomy 3 was the most frequent aberration seen in ABC DLBCL (26%). In cases with trisomy 3, *FOXP1* was the most frequently up-regulated gene. Indeed, high *FOXP1* expression is a characteristic finding of the ABC DLBCL subtype and *FOXP1* has been associated as an oncogene [12,34,35]. In total, 38% of ABC DLBCL had an increase in *FOXP1* CNA compared to 4% and 3% for GCB DLBCL and PMBL each. Another subtype specific lesion for ABC DLBCL included *NFKBIZ* identified in a chromosome 3 amplicon. It was detected in 9% of the cases, while never identified in GCB DLBCL or PMBL. *NFKBIZ* activates targets in the nuclear factor *kB* pathway a hallmark for ABC DLBCL [36]. In terms of the gain/amplification of 18q, which was significantly more frequent in ABC DLBCL than the other molecular subtypes*, BCL-2* and *NFATC1* were consistently up-regulated by the gain/amplification of 18q. Another distinctive feature of ABC DLBCL compared to the other two molecular subtypes was deletion of the *INK4a/ARF* tumor suppressor locus; 30% of the ABC DLBCL cases were deleted compared to 4% in GCB DLBCL and 6% in PMBL. This locus encodes for three tumor suppressors*: CDKN2A* (p16), *CDKN2B* (p15) and p14 *ARF*. For gain/amplification of 19q, the overexpression of the *SPIB* gene seems to play a more functional role in the pathogenesis of ABC DLBCL. Recent work has revealed a translocation between *SPIB* and the immunoglobulin heavy chain at 14q32 in ABC DLBCL [37]. Aberrations seen in GCB DLBCL included amplification of the mir-17-92 microRNA in the *MIHG1* locus cluster on chromosome 13, which has been shown to collaborate with *MYC* to transform B-cells and to reduce apoptotic activity [38]. The 1.4-Mb amplified region on chromosome 13 was detected 12.5% of the time in GCB DLBCL, rarely in PMBL (3%), and not observed in ABC DLBCL. A gain of a 7.6-Mb region on chromosome 12 revealed up-regulation of *MDM2*, a negative regulator for the p53, while deletion of the *PTEN* tumor suppressor gene on chromosome 10, and amplification of the *REL* locus on chromosome 2 were also more common in this molecular subtype. Interestingly, cases with *PTEN* deletion had a t(14;18) translocation suggesting that loss of *PTEN* may play a significant role in the pathogenesis of GCB DLBCL with t(14;18). The most frequent chromosomal lesions seen in PMBL included amplifications of a telomeric region of chromosome 9p, monosomy 10, and gain/amplification of chromosome 20p. It is unclear what genes in these regions are functionally important in the pathogenesis of PMBL. Of note, genes that were more frequently up-regulated in these regions were *JAK-2* and T-cell inhibitor ligand *PD-L2*. These results confirm that DLBCL can be confidently sub-grouped based on array CGH results and gene expression profiles. In another study by Tagawa et al. [22], array CGH was used to further study the aberrations associated with ABC DLBCL and GBC DLBCL. Based on their results, the ABC DLBCL group was genomically characterized by more frequent gains of 3q23-q28, 18q11.2-q23, 19q13.41-q13.43 and loss of 6q22.31-q24.1 and 9p21.3, while the GCB group was genomically characterized by more frequent gains of 1q21.1-q23.3, 1q31.1-q42.13, 2p15-p16.1, 7q22.1-q36.2, and 12q13.1-q14. These results suggest that ABC DLBCL and GCB DLBCL are genetically distinct from one another and arise from separate genetic pathways [2] Table 1.

**Table 1:** Genomic Gains and Losses in ABC and GCB-DLBCL Molecular Subtypes

**Chromosome breakpoints**	**GCB DLBCL**	**ABC-DLBCL**	**Reference**
1q21.1-q23.3 G	G		^16^
1q31.1-q42.13	G		^16^
2p15-16.1	G		^16^
3		G	^16^
3q23-q28		G	^16^
6q		L	^16^
6q22.31-q24.1		L	^2^
7q22.1-q36.2	G		^16^
9p21.3		L	^16^
12q13-q14.1	G		^16^
18q		G	^16^
19q13.41-q13.42		G	^16^

Key: G, genomic gain; L, loss of genetic material.

### Large B-cell lymphoma specified by site

#### Primary mediastinal large B-cell lymphoma

Primary Mediastinal Large B-cell Lymphoma (PMBL) arises in the thymus from thymic B-cells that presents as a mass in the mediastinum [1] and represent a distinct entity within the germinal center (GC) derived high grade DLBCL [39]. Although classified as a subtype of DLBCL, it is noteworthy to mention that PMBL and classical Hodgkin lymphoma (HL) share remarkably similar molecular profiles, as well as certain clinical and histological features [19,40]. In efforts to better define PMBL, Palanisamy et al. [39] using classical CGH characterized PMBL by whole chromosome gains of 12, 21, 22 and whole chromosome loses of 11, 13, and 18. Also, frequent gains of 4q, 9q, 10p, 17p, 19p, 20q, 21q, 22q and losses of 3q, 7p, 8q, 9p, 11p, 11q, 13q, 18p, 18q, Xp, and Xq were seen in PMBL patients. Subsequent studies used array based CGH to document amplification of band region 9p24.1 in PMBL cell lines [40], and similar studies reported amplifications to 9p and 2p involving the *REL* locus on PMBL [21,40-42]. In 2007, Wessendorf et al. [43] further outlined chromosomal aberrations in PMBL using aCGH ( n = 37). Here, genomic gains of 9p24 (68%), 2p15 (51%), 7q22 (32%), 9q34 (32%), 12q (30%) and 18q21 (22%) were reported. Interestingly, this study also described 17 chromosome regions with genomic losses in more than 10% of the cases. In terms of clinical outcome, PMBL is now more favorable due to intensive chemotherapy and radiation therapy [44]. As stated previously, PMBL and cHD share very similar molecular profiles, for example, frequent amplifications at 9p24 have been reported in both PMBL and classical HL; however, it is of interest to note that a study by Feys and colleagues using cHD cell lines [45] reported deletions of chromosome 15q26.2 encompassing *RGMA* and *CHD2*, *STAT6* up-regulation and deletion at 16q12.1 that were not found to occur in this current review of PMBL. Further investigation is needed to see whether these regions can be used to distinguish PMBL from cHD.

#### Large B-cell lymphoma of the bone (not listed as an entity in 2008 WHO)

Large B-cell lymphoma of the bone (LBCLB) is a subtype of primary extra nodal DLBCL. It typically presents in the longer bones, such as the humerus, tibia, pelvis, spine or the femur. Patients present with pain, a palpable mass or fractures. Complete remission is frequently obtained with a combination of chemotherapy and radiotherapy [9,46]. In 2010, Heyning et al. [46] used array-CGH to study nine primary lymphoma of the bone. Aberrations that were frequently seen included loss of 1p35-36.3, 6q14-27, 14q32, 15q11-26, trisomy 7, gain of 1q21-44, 6p21 and amplification of 2p16.1. Eight of nine patients reached complete remission in this study. These results support previously described GC-like properties of LBCLB, particularly that of better clinical outcome, 1q gain and 2p16.1 amplifications.

#### DLBCL of the central nervous system

DLBCL of the CNS represents a subtype that comprises all primary intracerebral or intraocular lymphomas [1]. Booman et al. reported genomic aberrations associated with DLBCL of the CNS using an array-based CGH (n = 9). The most common genomic aberrations seen in this study were loss of 6p21.32-p25.2 (56%), 6q (56%), 17p12-p13.3 (56%) and gains of 1q21.3-q32.1 (33%), 12(44%), 15q12-q21.1 (22%), 7/7q (22%), 18q (22%) and 19q13.12-q13.43 (22%) [47]. A similar study by Montesinos et al. [48] reported similar findings. DLBCL of the CNS is predominately of the molecular ABC subtype, thus explaining the poor prognosis of this subset of DLBCL [49].

#### Primary cutaneous LBCL, leg type

Based on the WHO classification scheme, there are three types of primary cutaneous B-cell Lymphoma: leg type (PCBCL), follicular center, and marginal zone. Leg type is a DLBCL that presents with large transformed B-cells that commonly arise in the leg at first [1]. In One study [50], using array CGH and fluorescent in-situ hybridization on 6 well characterized cases of PCBCL reported distinct genomic aberrations. This study showed recurrent gains of 1p36.33, 3p21.3, 7p, 7q11.21, 7q21.1, 11q13, 12q12-q13, 17q11.2, 17q21-22, 18q11.2, 18q21.1, 19q and losses to 9p21, 6q22-q23 and 17p11.2-p12 in 50% or more of the cases. Among the chromosome regions with recurrent gains found in at least 33% of the cases included: 1q25-q31, 1q41, 1q, 2p22, 2p12-q11, 3p21-p25, 3q, 7q, 8q24, 9p12-q21, 11q, 12p11, 12q13-q15, 12q32, 16q23, 17q11-q12, 17q21, 18q, 19q13, 20q13,22q11 and 22q13. Similarly, recurrent losses in 33% of the cases were 1p36.31, 1p31-p32, 1p13, 4q, 6q, 8p, 8q11, 9p11, 14q, Xq13 and Xq25. One of the most frequent aberrations mentioned in this study was the loss of 9p21(83%). Of the six patients with 9p21 deletion, all died in this study. Similar observations have reported deletions to 9p21.3, for example, one study identified 9p21 loss in eight of 12 patients with PCBCL [51]. Of the seven patients that died in this study, five cases had a deletion at 9p21. This may suggests that loss of this region has important clinical significance. In a recent study, it was revealed that the incidence of 9p21 loss was more prevalent in the ABC molecular subtype compared to the GC DLBCL subtype, demonstrating a poor clinical outcome for the loss of 9p21 [23]. Indeed, PCBCL is associated with the ABC DLBCL molecular subtype and requires therapy intensification [50].

### Large B-cell lymphoma specified by histology, phenotype, or genotype

#### T-cell Histiocyte-rich B-cell lymphoma

T-cell/histiocyte-rich B-cell lymphoma (T/HR) has been categorized as a DLBCL, but it had been done with much controversy due to the ambiguous presentation of the malignancy. T/HR LBCL is distinguished by a few scattered, large, atypical B-cells surrounded by a large quantity of T-cells and scattered histiocytes [1]. Classical CGH studies by Franke et al. [52] revealed most common gains of 4q13q38,18q21, Xq and Xp21-p11, as well as recurrent losses 17p. Most frequent CNA in this above report were Xq12-13 (58%), 4q25-q26 (41%), Xp11-21 (29%), 18q21 and 17p (24% each). Molecular profiles have identified most T/HR DLBCL cases to a subgroup of DLBCL distinguished by a “host response” with an adverse clinical outcome [53]. To the best of our knowledge, there is no array based CGH reports on this entity.

#### De novo, CD5+ large B-cell lymphoma

DLBCL expresses a variety of B-cell surface markers including CD5 in approximately 10% of cases [54]. Previous studies have reported a worst clinical outcome for CD5+ DLBCL compared to CD5- DLBCL [55,56]. In a genome wide array based CGH (n = 25), Tagawa et al. [57] identified genomic gains in more than 30% of the case in the following band regions: 1q23.1, 3q12.1, 3q27.3, 11q23.3, 11q24.3, 12p12.1, 12q13.3, 12q14.1, 12q15, 13q21.32, 13q32.3, 16p13.3, 18q21.1, 18q22.3, 19q13.33, 19q13.41, 19q13.43. Likewise, genomic losses were detected in 1p36.32, 6q21, 8p23.3, 9p21.3 and 17p13.1. Of note, by concentrating on the gain of 13q21-34 and loss of 1p34-36, Tagawa et al. was also able to recognize prognostically distinct subgroups within the CD5+ DLBCL subset. Moreover, in a separate study [23], Tagawa et al. identified 3q23-3q28 (31%), 6q22.31-q24.1 (44%) and 9p21.3 (50%) in high frequency for CD5+ DLBCL. When comparing CNA and clinical outcome, this latter study demonstrated 9p21 marked the most aggressive cases. In fact, Kreisel et al. [27] showed 9p21.3 band region as chemoresistant in DLBCL.

### Large B-cell lymphoma (LBCL) associated with epstein-barr virus and/or kaposi sarcoma–associated herpesvirus/human herpesvirus 8

#### DLBCL associated with chronic inflammation

Diffuse large B-cell lymphoma associated with chronic inflammation (CI) is a DLBCL associated with long lasting inflammation that is associated with EBV+. In most cases, this subtype of DLBCL develops in small body cavities and narrow spaces [1,10].

#### Pyothorax-associated lymphoma

Pyothorax-associated Lymphoma (PAL) develops in the pleural cavity of patients with a history of long-standing pyothorax [1]. Most reported cases of PAL have occurred in Japan, with few cases reported in western countries. Immunoglobulin genes in PAL are usually clonally rearranged and usually *TP53* mutations can be seen [58,59]. PAL is usually a precursor to DLBCL associated with chronic inflammation [10]. Few studies have attempted to define distinct CGH profiles for the PAL subtype. Using classical CGH analysis, Yamato et al. [60] showed the amplification of the 8q24 band region in 7 PAL cases. Amplification was later confirmed in four cases by southern blot analysis. Another separate study reported an over-expression of the interferon alpha-inducible protein 27, *IFI27*[61]*.* By cytogenetic analysis, one study reported complex karyotypes but no common abnormality was reported [62].

#### Plasmablastic lymphoma

Plasmablastic lymphoma (PL) is a scattered proliferation of large neoplastic cells that have the immunophenotype of plasma cells but resemble B-immunoblasts. It was originally described in the oral cavity but may occur in other extra-nodal sites [1]. In 2009, an array-based CGH conducted by Chang et al. [63] reported gains (>40%) of 1p36.11-1p36.33, 1p34.1-1p36.13, 1q21.1-1q23.1, 7p21.3-7p23 (38%), 7q11.2-7q11.23, 8q24.3 (25%), 10p12 (23%), 11q12-11q13.2, 14q32 (31%), 16p13.2-p13.3 (38%), 16q24 (38%), 17p13 (38%), 20q11.1-q11.23 (38%) and 22q12.2-22q13.3 in PL, while genetic losses were more diverse. However, only 1p35.1-1p36.12, 1q21.1-1q23.1 and 1p36.11-1p36.33 were unique to PL when compared with DLBCL (AIDS related and non-AIDS related) and plasma cell myeloma. The clinical outcome for these patients is poor [64].

#### Primary effusion lymphoma

Primary Effusion Lymphoma (PEL) is another rare subtype of DLBCL that presents as an extravascular collection of fluids with no identifiable tumor mass. It is associated with the Human Herpes Virus 8 (HHV8 +), the Kaposi sarcoma herpes virus (KSHV) and carries a poor clinical outcome in PEL patients [1,65,66]. Very little is known about the genetic aberrations associated with this malignancy. A recent array CGH study revealed gains of 1q21-41 (47%), 4q28.3-35 (29%), 7q (58%), 8q (63%), 11 (32%), 12 (61%), 17q (29%), 19p (34%), 20q (34%) and losses of 4q (32%), 11q25 (29%) and 14q32 (63%) [67]. In an earlier study using classical CGH (n = 5), Ohshima et al. [68] identified gains of 3q13-q27, 8q24, 8, 10q21-23 and Yq in HHV8 negative PEL cases.

### Unclassifiable types

#### LBCL with features intermediate between DLBCL and Burkitt lymphoma

Large B-cell lymphoma with features intermediate between DLBCL and Burkitt lymphoma (INT) is an aggressive lymphoma that has overlapping features between DLBCL and Burkitt lymphoma. Approximately 35-50% of INT cases have *MYC* rearrangements [69,70] with a concurrent *BCL-2* translocation (“double-hit lymphoma”) in approximately 15% of cases. A limited number of array based CGH studies are available. In 2006, Hummel et al. [69] using aCGH reported most of these cases with a high chromosomal complexity with a score of 6 or more abnormalities with a *MYC* rearrangement.

### DLBCL subtypes with limited or no array-based CGH information

#### Intravascular large B-cell lymphoma

Intravascular Large B-cell lymphoma (ILBCL) is differentiated by localization of atypical lymphomatous cells within smaller vessels and capillaries [1,71]. It is a rare, aggressive extra nodal B-cell lymphoma that is recognized in the 2008 WHO classification as a subset of DLBCL. ILBCL is known as the great imitator due to the fact that it presents with a broad spectrum of nonspecific symptoms with no distinct array CGH profile [10].

#### Anaplastic lymphoma kinase-positive LBCL

Anaplastic large cell lymphoma with expression of the anaplastic kinase protein is a rare neoplasm of ALK-positive monomorphic large B-cells with immunoblastic or plasmablastic morphology. The only chromosome aberration associated with this disorder is the involvement of the *ALK* gene on chromosome 2. The gene is usually involved with the following translocations: t(2; 17)(p23;q23) or t(2:5)(p23;q35). This type of lymphoma is a rare entity constituting only less than 1% of DLBCL cases. Thus, no substantial CGH data is available [1,10]. Patients are unresponsive to rituximab and have a poor clinical outcome [72].

#### EBV + DLBCL of the elderly

Epstein-Barr virus-positive DLBCL of the elderly is a newly recognized subtype of DLBCL by the WHO classification (2008) which presents as an EBV + clonal B-cell proliferation usually in patients over 50 years [1,73,74]. EBV + DLBCL is such a new classification of DLBCL that there is not sufficient CGH array data to create a genomic profile or distinctive cytogenetic pattern for such malignancy [75].

#### Large B-cell lymphoma arising in HHV8+ multicentric castleman disease

LBCL occurring in HHV8+ associated multicentric castleman disease is characterized by monoclonal proliferation of HHV8+ lymphoid cells in the presence of multicentric castleman disease [1]. It is an aggressive disorder with no information about the cytogenetics or CGH profiles [1,76].

#### Lymphomatoid granulomatosis

Lymphomatoid Granulomatosis (LG) is an angiocentric and angiodestructive proliferative B-cell neoplasm that involves extra-nodal sites. LG is composed of malignant B-cells that are Epstein-Barr virus-positive combined with reactive T-cells. Clinical outcome is variable and depended on the quantity of large B-cells [1]. The majority of studies on LG have failed to identify unique chromosomal aberrations; however, a study by Godde-Salz et al. [77] reported few consistent chromosome aberrations including trisomy of chromosome 3 and 5 along with duplications of the X chromosome.

#### LBCL with features intermediate between DLBCL and Hodgkin lymphoma

This type of lymphoma is characterized by clinical and morphological features that overlap between DLBCL, particularly primary mediastinal large B-cell lymphoma, and classic Hodgkin lymphoma. Only limited cases of LBCL with features intermediate between DLBCL and Hodgkin lymphoma have been studied.

**Figure 1:**
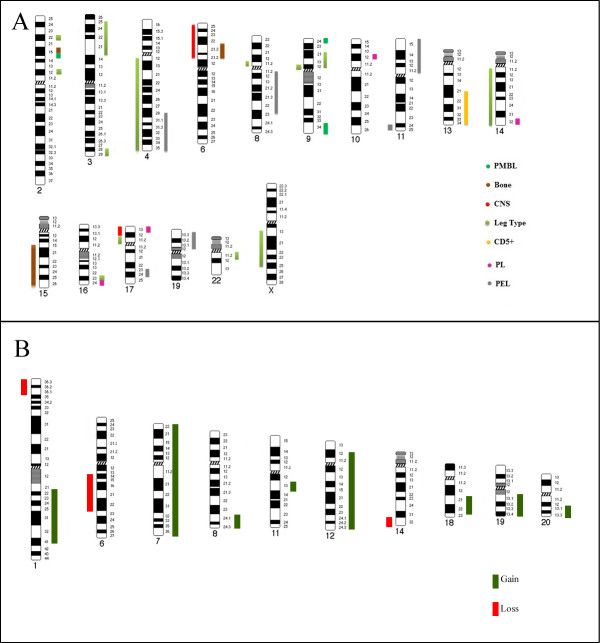
**Panel A shows an ideogram spectrum for unique gains and losses for the various DLBCL subtypes.** Chromosome band region gains are provided on the right hand side of each chromosome, while genetic losses are shown on the left hand side of each chromosome ideogram. Each category is represented by a different color coded bar as illustrated in the schematic. **Panel B** illustrates common CNA for all subtypes. Commonalities were highlighted if three or more subtypes had a CNA at that particular band region.

The table below summarizes genomic gains and losses for the various DLBCL subtypes, Table 2-, Figure 1-.

**Table 2:** Array CGH data of gains and losses for the various DLBCL Subtypes

**Chromosome breakpoints**	**PMBL**	**Bone**	**CNS**	**Leg type**	**T/HR**	**CD5+**	**PAL**	**PL**	**PEL**
1p36		**_**		**+/−**		**_**		**+**	
1p35-p34		**_**						**+**	
1q				**+**					
1q11				**+**					
1q21		**+**	**+**	**+**				**+**	**+**
1q22		**+**	**+**	**+**				**+**	**+**
1q23		**+**	**+**	**+**		**+**		**+**	**+**
1q24-q25		**+**	**+**	**+**					**+**
1q31-q32		**+**	**+**	**+**					**+**
1q36		**+**		**+**					**+**
1q41		**+**		**+**					**+**
1q43-q44		**+**		**+**					
2									
2p22				**+**					
2p16		**+**							
2p15	+								
2p12				**+**					
2p13									
2p11									
2q33									
3									
3p21-p25				**+**					
3p21				**+**					
3p14									
3q				**+**					
3q12-q24						**+**			
3q25						**+**			
3q26-q27						**+**			
4q28-35									**+**
4q				**_**					**_**
5									
5p15									
5p13									
5q11-q31									
6									
6p25-p22			**_**						
6p21		**+**	**_**						
6q			**_**	**_**					
6q14-q21		**_**	**_**	**_**		**_**			
6q22		**_**		**_**		**_**			
6q23-q27		**_**		**_**					
7		**+**	**+**						
7p				**+**					
7p21				**+**				**+**	
7q			**+**						**+**
7q11			**+**	**+**				**+**	**+**
7q21			**+**	**+**					**+**
7q22	**+**		**+**	**+**					**+**
7q31			**+**	**+**					**+**
7q32			**+**	**+**					**+**
8									
8p				**_**					
8p11				**_**					
8p23-p21				**_**		**_**			
8q									**+**
8q24				**+**			**+**	**+**	**+**
9									
9p11				**_**					
9p24	**+**								
9p12-21				**+**					
9p21				**_**		**_**			
9q34	**+**								
10q									
10p									
10p12								**+**	
10q21-q23									
11									**+**
11p									
11q				**+**					
11q12								**+**	
11q13				**+**				**+**	
11q21-22									
11q23-24						**+**			
11q24-q25									**_**
12			**+**						**+**
12p11-12				**+**		**+**			
12q	**+**								
12q12	**+**			**+**					
12q13-q23	**+**			**+**		**+**			
12q24	**+**								
13									
13p11									
13q									
13q21-q31						**+**			
13q32						**+**			
13q33q34						**+**			
14									
14q				**_**					
14q32		**_**		**_**				**+**	**_**
15									
15p11									
15q		**_**							
15q12		**_**	**_**						
15q13-q14		**_**	**_**						
15q22		**_**							
16									
16p13						**+**		**+**	
16q									
16q11									
16q12									
16q13									
16q23				**+**					
16q24								**+**	
17									
17p									
17p13-p12			**_**					**+**	
17p11.2-p12				**_**					
17q11				**+**					**+**
17q21				**+**					**+**
17q23-q24									**+**
18									
18p									
18q			**+**	**+**					
18q21-22	**+**		**+**	**+**		**+**			
19q				**+**					
19p									**+**
19p13									+
19q13			**+**	**+**		**+**			
20q/20q13				**+**				+	**+**
21									
21q									
22q11				**+**					
22q12-q13				**+**				**+**	
X									
Xp11.4-21	**+**								
Xq13/q25									
Xq24-26	**+**			**_**					
Yq									

Key: +, Gain; -, Loss; PMBL, Primary mediastinal large B-cell lymphoma; Bone, Primary large B-cell lymphoma of bone; CNS, DLBCL of the central nervous system; Leg type, Primary cutaneous large B-cell lymphoma leg type; T/HR, T-cell/histiocyte-rich B-cell lymphoma (no aCGH data available); CD5+ , De novo CD5 large B-cell lymphoma; PAL, Pyothorax-associated lymphoma; PL, Plasmablastic lymphoma; PEL, primary effusion lymphoma. Amplification of 8q24 listed here in PAL was detected by classical CGH and southern blot analysis.

## Conclusions

In this review, we explored associated copy number alterations (CNA) in 2008 WHO-defined DLBCL subtypes by array CGH. Certain chromosomal aberrations that were significantly more frequent in a particular DLBCL subtype than in the others, and some of these aberrations were associated with clinical outcome. Following our review of aCGH microarray studies, a number of subsets were re-classified, for example, unique chromosome loci were identified in the following subtypes: PMBL, gain at 2p15, 9p24, 9q34, Xp11.4-21, Xq24-26; Large B-cell lymphoma of the bone, gain at 2p16, 6p21 and loss at 15q15-q26; DLBCL of the CNS, loss at 6p21-25,17p12-13; Leg type DLBCL, gain at 2p22, 2p12, 3p21-25, 3q28-29, 9p12-21,16q23,22q11and loss at 4q, 8p11,14q, Xq13-25; CD5+ DLBCL, gain at 13q21-34; Plasmablastic lymphoma, gain at 10p12, 14q32, 16q24,17p12-13 and primary effusion lymphoma with gains at 4q28-35, 8q11.2-23.1, 11p, 17q23-24, 19p13 and loss at 11q24-25. However, despite these efforts, there is still a number of unclassifiable DLBCL subtypes post-microarray studies. Among these include intravascular LBCL, EBV + DLBCL of the elderly, large B-cell lymphoma arising in HHV8+ multicentric Castleman disease, Lymphomatoid granulomatosis, LBCL with features intermediate between DLBCL and HL and PAL. Several reasons for this may include: a limited number of study cases, uncommon disease entities and a comparatively small number of publicly available aCGH datasets. Therefore, future studies should aim on the copy number alterations in newly defined uncommon large B-cell lymphoma entities, such as EBV + DLBCL of the elderly, ALK positive DLBCL and LBCL arising in HHV8-associated multicentric castleman disease. Moreover, as more array based CGH datasets become publicly available, meta-analysis studies should further characterize DLBCL subsets. Likewise, given the large collective number of gene expression datasets for DLBCL subtypes and using novel computational methods such as hidden Markov models to predict CNA from gene expression profiling [78] should significantly improve our understanding of the biology, clinical outcome and therapeutic management of DLBCL. Moving forward, microarray analysis should be used in an integrative approach using aCGH, gene expression profiles, SNP arrays and next generation sequencing techniques to better categorize DLBCL subtypes.

In short, our analysis provides a rich starting point for future investigations into the molecular pathogenesis of DLBCL. This review revealed oncogenic pathways that are used differentially by the DLBCL subtypes, reinforcing the view that they represent pathogenetically distinct diseases.

## Competing interests

The authors declare that they have no competing interests.

## Authors’ contributions

CAT Lead the whole manuscript writing WC also wrote and did a review of the literature KK did the tables and drafted the first version of the manuscript. RG did the hard work of interpreting the tables and he also created the figures NR revised everything. All authors read and approved the final manuscript.
